# Anesthetic Management of a Patient With Distal Acquired Demyelinating Symmetric (DADS) Neuropathy Under General Anesthesia

**DOI:** 10.7759/cureus.27417

**Published:** 2022-07-28

**Authors:** Stefen P Roth, Thomas Gemma, Tracy Dovich, Anthony Candella

**Affiliations:** 1 Transitional Residency, Clarion Hospital, Clarion, USA; 2 Anesthesia, The Surgical Hospital at Southwoods, Youngstown, USA

**Keywords:** demyelinating diseases, basic and advanced training in anesthesiology, anesthesiology, anesthetic management, chronic inflamatory demyelinating polyneuropathy

## Abstract

Distal acquired demyelinating symmetric (DADS) neuropathy is a form of chronic inflammatory demyelinating polyradiculopathy (CIDP) which can present many risks to patients undergoing anesthesia. There are currently no specific guidelines for the management of patients with any form of CIDP. This case report describes a 65-year-old male with DADS neuropathy who underwent elective total hip arthroplasty. General, total IV anesthesia was used in combination with other short-acting drugs and the patient endured a successful procedure with an uneventful post-operative course. This case may provide future insight into the anesthetic management of patients with similar comorbidities.

## Introduction

Distal acquired demyelinating symmetric (DADS) neuropathy is a form of chronic inflammatory demyelinating polyradiculopathy (CIDP) that is characterized mainly by chronic, distal sensory, or sensorimotor deficits [1]. Many chronic neuropathies are characterized based on their specific phenotypes, whereas most patients with DADS present with immunoglobulin M (IgM) Kappa monoclonal gammopathies and antimyelin-associated glycoprotein (anti-MAG) antibodies. Patients may experience a relapsing-remitting disease course involving distal sensory deficits and motor weakness even when properly managed. CIDP's rarity means that there are currently no standardized anesthetic management protocols for the disease. Here, we present a case report of successful anesthetic management of a patient undergoing elective total hip arthroplasty.

## Case presentation

The patient is a 65-year-old male (178 cm and 105.7 kg) with a past medical history of DADS neuropathy, mild chronic obstructive pulmonary disease (COPD), and hyperlipidemia who underwent a left total hip arthroplasty at an elective surgical center due to osteoarthritis. No significant family history exists relating to his DADS neuropathy which dates to 2015, beginning as bilateral symmetric paresthesia in his feet. In addition, the patient also began to develop bilateral weakness and heaviness ascending up his legs. He was later tested and found to have an M protein spike of 0.3 g/dL with detectable levels of anti-MAG antibodies. The diagnosis of DADS neuropathy was confirmed with electromyography. Early in 2016, the patient felt that the paresthesia and weakness were ascending into his thighs, and therefore he began treatment with monthly, three-dose intravenous immune globulin (IVIG), and biannual rituximab was given once a week for four weeks. The patient's neuropathy improved with the administration of IVIG, which caused the pain to be confined only to his feet. He continues with the aforementioned treatments as maintenance therapy to this day.

A perioperative physical exam showed a pulse rate of 90 bpm, manual blood pressure of 116/70 mmHg, a temperature of 37°C, and a respiratory rate of 18 breaths/min while saturating 98% on room air. A neurological exam revealed intact cranial nerves with equal, round, and reactive pupils bilaterally. Upper extremity muscle strength was 5/5 with normal sensation and 2+/4 muscle stretch reflexes bilaterally. Lower extremities showed 5/5 strength, decreased sensation to pain and vibratory sensation below the ankle, and 1/4 Achilles tendon muscle stretch reflex bilaterally. No cerebellar signs were present. The patient was premedicated per Enhanced Recovery After Surgery (ERAS) protocol with 1,000 mg acetaminophen, 300 mg gabapentin, and started on IV fluids with Lactated Ringer’s. Approximately 15 min prior to induction, the patient was given 1 mg midazolam and 100 mcg fentanyl as a preoperative anxiolytic and analgesic, respectively. Induction began with 50 mg of lidocaine (suggested dose of 0.5-1.5 mg/kg for airway reflex suppression), 2000 mcg/kg bolus of propofol, 1 mcg/kg bolus of remifentanil (to aid in further suppressing airway reflexes and to more rapidly achieve steady-state plasma concentrations preceding maintenance), and 50 mg rocuronium followed by uncomplicated intubation. Bispectral Index (BIS) was utilized to monitor levels of consciousness and effectiveness of anesthesia, and therefore maintained between 40 and 50. The procedure lasted approximately two hours and anesthesia was maintained under total intravenous anesthesia (TIVA) using propofol at 80 mcg/kg/min. The patient was eventually reversed with 400 mg sugammadex and extubated when observed to be breathing adequately on his own. He was sent to the post-anesthesia care unit immediately following the procedure and admitted to the general floor overnight for observation. The patient was sent home on post-operative day one without any overnight complications.

## Discussion

Chronic inflammatory demyelinating polyradiculopathy is an autoimmune demyelinating disease with an unknown etiology that progresses and lasts over the course of at least eight weeks [2]. Its prevalence is estimated at around 1.0-8.9 per 100,000 people, occurring at any age, with a mean age onset of 47.6 years [2]. One of its main subtypes known as DADS neuropathy is characterized specifically by its distal-only sensory or sensorimotor deficits [1]. Patients often undergo long-term treatment with first-line agents such as corticosteroids, intravenous immunoglobulin, and plasma exchange therapy [2]. Due to the limited amount of current literature regarding anesthetic management in patients with forms of CIDP, this patient's surgery proceeded without any specific standardized guideline on how to manage the patient while under anesthesia.

Patients undergoing total hip arthroplasty may receive general or regional anesthesia. One concern in patients with CIDP undergoing regional blocks is that the anesthetic may spread too far cranially and affect respiratory muscles, which may already be weakened [3]. A previous case report conducted on a patient with CIDP undergoing cesarean section suggests that regional anesthesia, even if the dose is reduced, may prolong the recovery of motor function in these patients [4]. Another case report on anesthetic management options in patients with CIDP compares CIDP neuropathy to similar peripheral demyelinating syndromes such as Guillain-Barre syndrome (GBS) and Charcott-Marie-Tooth type 1 (CMTt1) in which more evidence does exist [5]. There does not seem to be an exacerbation of such diseases with regional anesthesia, but it is recommended to observe these patients very closely for adverse hemodynamic effects, as they may have greater sensitivity to local anesthetic toxicity on already demyelinated nerves.

Patients with autoimmune polyneuropathies undergoing surgical procedures also face the risks of post-operative respiratory complications and prolonged effects of muscle relaxants. In a prior case report published on a patient with CIDP undergoing surgery on the neck of the femur, the authors discussed that if administered, there is the possibility of regional anesthesia affecting nerves involving respiratory intercostal muscles, as well as possible uncertainty of the exact neuraxial block levels that may be involved [6]. Under general anesthesia, the use of depolarizing muscle relaxants has been associated with an increased risk of malignant hyperthermia and hyperkalemia in patients with GBS, possibly due to an overexpression of cholinergic receptors that release increased amounts of potassium [5]. Non-depolarizing agents, albeit drugs of choice in these patients, may cause prolonged muscle relaxation and should be used with caution. A more recently published case report from 2019 on the anesthetic management of patients with CIDP provides evidence of the successful use of rocuronium with sugammadex in patients with CIDP and GBS, although allergic reactions to sugammadex in some patients were reported [7].

Ultimately, we decided to manage the patient with general anesthesia to avoid any risk of exacerbating his disease with regional anesthesia, and to establish a secure airway via endotracheal intubation, as opposed to a laryngeal mask airway (LMA). The duration of the procedure, the potential for medication adverse effects, and the risks of respiratory complications played large roles in our consideration for general anesthesia. Additional consideration was given to general anesthesia due to the lesser amount of literature on maintaining a CIDP patient under any type of anesthesia as well. The promising literature on rocuronium paired with sugammadex provided us with further reassuring evidence, as our patient was given the minimum dose of rocuronium during induction followed by sugammadex to regain control of muscle strength before being extubated. His immediate postoperative course was uneventful, and he suffered no respiratory complications or insufficiencies before being discharged to his home on the next day.

## Conclusions

During this case, we managed a patient with DADS neuropathy, a form of CIDP, and mild COPD under general anesthesia during an elective hip replacement surgery. Short-acting agents were used in combination with opiates, rocuronium, and sugammadex to produce a successful and uncomplicated outcome. Although individual patient comorbidities should be considered on a case-by-case basis, we strongly encourage this approach in future practice and similar patient populations.
